# Assessment of Aortoiliac Atherosclerotic Plaque on CT in Prostate Cancer Patients Undergoing Treatment

**DOI:** 10.3390/tomography8020050

**Published:** 2022-03-01

**Authors:** Sungwon Lee, Daniel C. Elton, James L. Gulley, Perry J. Pickhardt, William L. Dahut, Ravi A. Madan, Peter A. Pinto, Deborah E. Citrin, Ronald M. Summers

**Affiliations:** 1 Imaging Biomarkers and Computer-Aided Diagnosis Laboratory, Department of Radiology and Imaging Sciences, National Institutes of Health Clinical Center, Bethesda, MD 20892, USA; sungwonlee8@gmail.com (S.L.); delton@mgh.harvard.edu (D.C.E.); 2 Genitourinary Malignancies Branch, National Cancer Institute, National Institutes of Health, Bethesda, MD 20892, USA; gulleyj@mail.nih.gov (J.L.G.); dahutw@mail.nih.gov (W.L.D.); madanr@mail.nih.gov (R.A.M.); 3 Department of Radiology, The University of Wisconsin School of Medicine & Public Health, Madison, WI 53726, USA; ppickhardt2@uwhealth.org; 4 Urologic Oncology Branch, National Cancer Institute, National Institutes of Health, Bethesda, MD 20892, USA; pintop@mail.nih.gov; 5 Radiation Oncology Branch, National Cancer Institute, National Institutes of Health, Bethesda, MD 20892, USA; citrind@mail.nih.gov

**Keywords:** prostatic cancer, atherosclerotic plaques, contrast-enhanced, cardiovascular diseases

## Abstract

Traditionally, atherosclerotic risk factors for cardiovascular disease and cancer are assessed using coronary artery calcium scoring. However, this neglects the impact of atherosclerotic disease more proximal to the cancer site. This study assesses whether aortoiliac atherosclerotic plaque is associated with prostate cancer. The dataset consisted of abdominopelvic CT of 93 patients with prostate cancer and 186 asymptomatic patients who underwent CT colonography as an age- and gender-matched control group. Agatston scores were measured in the abdominal aorta, common iliac, and internal iliac arteries. The scores were evaluated for associations with age, Framingham risk score, and prostate cancer-related biomarkers, including prostate-specific antigen, Gleason score, tumor location, prostatectomy, androgen deprivation therapy, mortality, and bone metastasis. The atherosclerotic plaque of prostate cancer patients did not differ from the control group (*p* = 0.22) and was not correlated with any of the prostate cancer-related biomarkers (*p* > 0.05). However, Agatston scores of abdominal plaques correlated well with age (*p* < 0.001) and Framingham risk scores (*p* = 0.002).

## 1. Introduction

Cancer survivors have a higher incidence of cardiovascular disease (CVD) compared to the general population [1], and increased coronary artery calcification has been observed in cancer-diagnosed and cancer-treated patients [2]. Prostate cancer patients also have a high incidence of CVD [3], and local atherosclerosis has been found in prostatectomy specimens of prostate cancer patients [4]. 

Abdominal aortic calcification is known to be correlated with traditional CVD risk factors, including age, gender, smoking, hypertension, and Framingham risk score [5,6,7]. However, the plaque burden in the abdominal aortic and iliac arteries of prostate cancer patients has yet to be reported. A technical challenge is that calcification scores are usually measured on non-contrast chest CT scans using the Agatson score (reflecting the total area of calcium deposits and density) [8], but in daily practice, most abdominal CT scans are performed with intravenous contrast enhancement. 

**Figure 1:**
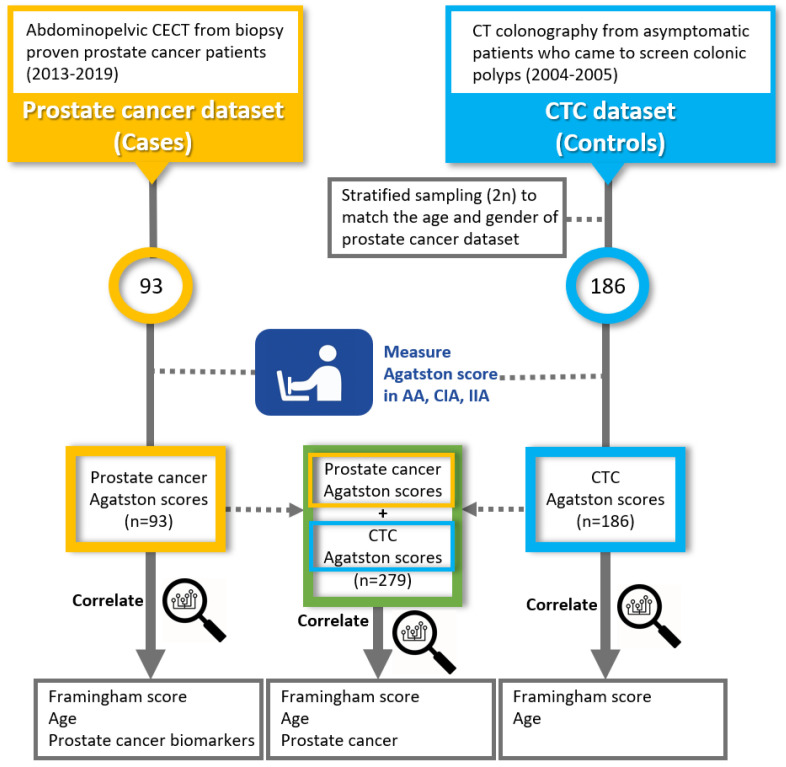
Flow diagram of study outline. Abbreviations: CECT, contrast-enhanced CT; CTC, CT colonography; AA, abdominal aorta; CIA, common iliac artery; IIA, internal iliac artery.

This case-control study involved comparing the plaque burden of a prostate cancer patient group with an age- and gender-matched control group of asymptomatic outpatients undergoing a screening CT colonography (CTC). The primary objective was to see if the abdominal Agatston scores had an association with a prostate cancer diagnosis, prostate cancer-related biomarkers, and the Framingham risk score (a score that estimates the 10-year risk of manifesting clinical CVD) [9]. A flow diagram of the study design can be found in Figure 1.

## 2. Materials and Methods

This investigation was Health Insurance Portability and Accountability Act compliant and approved by the Institutional Review Boards at (BLINDED), and by the Office of Human Subjects Research Protection at (BLINDED). All research was performed in accordance with relevant guidelines/regulations and the Declaration of Helsinki. The requirement for signed informed consent was waived.

### 2.1. Datasets

Abdominopelvic contrast-enhanced CT (CECT) exams were retrospectively recruited from biopsy-proven metastatic adenocarcinoma prostate cancer patients (Gleason score 6 and higher) who participated in clinical trials involving neoadjuvant treatments at a single institution (BLINDED) from 2013 to 2019. The scans were taken at the beginning of the trial and performed with portal venous phase (PVP) contrast enhancement. Patients with missing clinical data or CT images were excluded. A total of 93 CECT scans were obtained. This dataset will be referred to as the Cases dataset.

The control group consisted of asymptomatic outpatients who had CT colonography (CTC) screening for detecting colonic polyps at a different institution from April 2004 to March 2005 [6]. To compare with the prostate cancer dataset, we selected 186 patients (2 times the size of the prostate cancer dataset) out of 829 patients by stratified random sampling. The age and sex were matched with the prostate cancer dataset. At the time of the scan, none of the 186 CTC outpatients had been diagnosed or treated for prostate cancer. During long-term (median 8.2 years) follow-up, 2 of the 186 outpatients developed prostate cancer, 2 and 5 years after the scan. This dataset will be referred to as the Controls dataset.

All CT scans were cropped to have the same field of view (FOV) from the diaphragm to the pelvic brim and resampled to 3 mm section thickness.

### 2.2. Agatston Score Measurement

**Figure 2:**
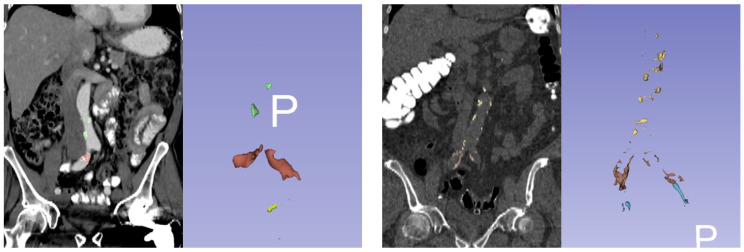
Example of Agatston score measurement. (**left**) A 72-year-old male with prostate cancer. Agatston scores for abdominal aorta (AA), common iliac arteries (CIA), and internal iliac arteries (IIA) were 752, 3932, and 506. (**right**) A 74-year-old asymptomatic male from the control group. Agatston score for AA, CIA, and IIA were 2814, 3265, and 1555. Plaques in AA, CIA, and IIA are colored differently. 3D images are facing anterior to posterior (P).

Agatston scores were determined as described in the Supplementary Materials. All plaques were segmented using the segment editor module of 3D Slicer (version 4.10 RRID: SCR_005619) and measured with plug-in Python codes modified from the ‘Cardiac Agatston Scoring function’ codes [10]. Plaque scores were measured separately in: 1. the abdominal aorta (AA: 1cm above the celiac axis to the bifurcation point of the common iliac arteries), 2. the common iliac arteries (CIA: bifurcation point of common iliac arteries to the bifurcation of the external and internal iliac arteries), and 3. the internal iliac arteries (IIA: bifurcation of the external and internal iliac arteries to the visible end arteries of the internal iliac arteries inside the pelvic cavity). All measurements were done by a single radiologist with 12 years of experience. An example of plaque measurement can be found in Figure 2.

### 2.3. Clinical Biomarkers

Framingham Risk Scores [9] were calculated from CVD risk factors, including age, gender, systolic blood pressure (at the time or closest to the time of CT scan), hypertension (diagnosed or currently being treated), smoking habits (divided into current or past/non-smoker), prior CVD events, diabetes (diagnosed or currently being treated), total cholesterol, and HDL cholesterol (at the time or closet to the time of CT scan) in the Cases and Controls datasets. Prostate cancer-related biomarkers including prostate-specific antigen (PSA) at the time of diagnosis, pathology (Gleason score, anatomical location of the tumor within the prostate gland), prostatectomy at the time of CT scan, androgen deprivation therapy (ADT) usage at the time of the scan, and patient outcomes were obtained in the Cases dataset. The outcomes were mortality and bone metastasis reviewed for a mean period of 23 ± 16 months.

### 2.4. Statistical Analysis

The Agatston scores of Cases and Controls were compared with the Wilcoxon signed-rank test. The AA, CIA, and IIA scores were compared separately. To determine if plaque appearance in a certain vessel correlated with plaques in other vessels, we also calculated the Spearman’s rank correlation coefficient between the Agatston scores of the AA, CIA, and IIA. Since the vessels had a different range of scores depending on the vessel size, the scores were first stratified as percentiles (zero, 25th, 50th, and 75th) before the Spearman correlation. 

To correlate clinical biomarkers with the Agatston scores, Cases and Controls were first compared separately with age and Framingham risk scores using Spearman’s rank correlation. Then, age, Framingham risk scores, and prostate cancer diagnosis were assessed by univariate and multivariate analyses for predicting the plaque scores in the combined prostate cancer and CTC datasets. The log transformation of Agatston scores was used to make the Agatston score variable more suitable for fitting linear models. Only the factors that had a *p*-value lower than 0.05 were put into the multivariate analysis. Residual plots and variance inflation factor (VIF) scores were used to examine the appropriateness and multicollinearity of the regression models. The prostate cancer-related biomarkers were compared with Agatston scores using univariate/multivariate analysis, Spearman’s rank correlation, Mann–Whitney U test, and Kruskal–Wallis test, according to data types. All statistics were computed using R (version 3.6.3 RRID: SCR_001905).

## 3. Results

### 3.1. Patient Characteristics

The patient characteristics and CT parameters of each dataset are shown in Table 1 and Table 2. There was no significant difference in patient age between the Case and Control datasets (Figure S1).

### 3.2. Agatston Scores of the Cases and Controls Patients

**Figure 3:**
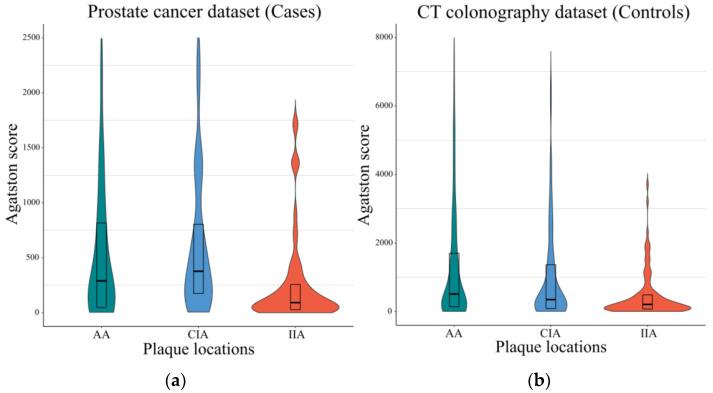
Agatston scores of Cases and Controls patients in different arterial territories. (**a**) Agatston scores of Cases patients in different vessels. The median scores were 340 (IQR, 60-930) for the abdominal aorta (AA), 407 (IQR, 178-1064) for the common iliac arteries (CIA), and 92 (IQR, 28-257) for the internal iliac arteries (IIA). Cases scores are before calibration. Only non-zero plaques are included. The violin plots display the full distribution of the data, and the box plots represent the median, upper, and lower quartiles. (**b**) Agatston scores of Controls patients in different vessels. The median scores were 555 (IQR, 152-2212) for the AA, 349 (IQR, 89-1364) for the CIA, and 208 (IQR, 76-486) for the IIA. Abbreviations: IQR, interquartile range.

Zero and non-zero Agatston scores were counted separately. In the Cases, the percentages of zero Agatston scores were 25.8% in the abdominal aorta (AA), 37.6% in the common iliac arteries (CIA), and 33.3% in the internal iliac arteries (IIA). In the Controls, the percent of zero Agatston scores were 16.3% in the AA, 26.3% in the CIA, and 31.9% in the IIA. The non-zero Agatston scores are shown in Figure 3.

**Figure 4:**
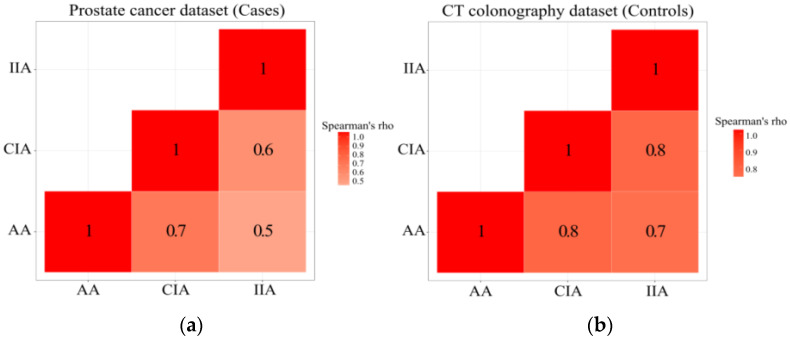
Correlation of Agatston scores between different vessels. (**a**) Correlation of Agatston scores between the abdominal aorta (AA), common iliac arteries (CIA), and internal iliac arteries (IIA) in Cases dataset. (**b**) Correlation of Agatston scores between the AA, CIA, and IIA in the Controls dataset. The scores were first stratified into quartiles before Spearman’s correlation.

In the Cases dataset, there were high correlations between the Agatston scores of the AA, CIA, and IIA (Spearman’s Rho ≥ 0.5). The Controls dataset also showed similar high correlations between the Agatston scores of the AA, CIA, and IIA (Spearman’s Rho ≥ 0.7). Correlation heatmaps can be found in Figure 4. 

When comparing the calibrated Cases and Controls scores, there was no statistically significant difference between the two groups in the AA, CIA, IIA, and the sum of all plaques (Wilcoxon *p* = 0.062, 0.59, 0.47, and 0.22, respectively).

### 3.3. Association of Plaque and Cardiovascular Disease (CVD) Risk Factors

Age and Framingham scores were correlated with the Agatston scores of both Cases (*p* <0.001, Spearman’s Rho (ρ) 0.37 for age, *p* <0.001, ρ = 0.41 for Framingham risk score) and Controls (*p* <0.001, Spearman’s ρ = 0.67 for age, *p* <0.001, ρ = 0.49 for Framingham risk score). This was true for the AA, CIA, IIA, and the sum of all plaques (Table 3). When age, Framingham scores, and prostate cancer diagnosis were applied to the 2 groups in combination, only age and Framingham score had a significant association in the univariate analysis (*p* < 0.001 for age, <0.001 for Framingham score) and multivariate analysis (*p* < 0.001 for age, 0.002 for Framingham score). Prostate cancer diagnosis had no association in the univariate analysis (*p* = 0.95) (Table 4). The VIF for age and Framingham scores were 1.33 and 1.33, respectively, and the residual plot for the multivariate analysis model had a symmetrical distribution (Figure S2).

### 3.4. Association of Plaque and Prostate Cancer-Related Biomarkers

There was no significant association of the PSA, Gleason score, mortality, bone metastasis, prostatectomy, ADT use, and tumor location with the Cases’ Agatston scores (Table 5).

## 4. Discussion

In this study, we found that the Agatston scores of the abdominal aortic and iliac plaques were not significantly different in the prostate cancer and control groups and also were not associated with prostate cancer-related biomarkers within prostate cancer patients. However, the abdominal aortic and iliac plaques had a good association with age and Framingham risk score, and this pattern was similar in both groups. We also examined the regional distribution of plaques and found that the abdominal aortic, common iliac, and internal iliac plaques were highly correlated with each other. This finding suggests that abdominal aortic plaque measurement alone may be sufficient in future investigations, increasing the efficiency of conducting such studies.

Prostate cancer and CVD have many shared etiologies, which adds complexity to explaining study results. Most published works that study CVD in prostate cancer patients focus on the coronary arteries, a more systemic marker. Abdominal arteries are more proximal to the site of prostate cancer and might theoretically have a closer association with the presence of cancer. In addition, many studies reporting a correlation between CVD and prostate cancer are not free from screening biases (higher screening rate of patients with underlying CVD) or confounding factors such as age, obesity, and cholesterolemia [3]. In a longitudinal coronary calcium score study, men with higher coronary calcium scores had an increased chance of developing cancer (55% prostate cancer) [2]. However, after adjusting for age, there was no significant difference between the cancer group and cancer-free group. Although other possible confounding factors exist (e.g., obesity and cholesterol), the prostate cancer group and control group in our study were age-matched and also showed no difference in the abdominal aortic and iliac calcifications. It is possible that the presence of stenoses, particularly in the smaller internal iliac arteries, may be associated with prostate cancer. This hypothesis could form the basis of future research.

ADT treatment of over 6 months is also reported to contribute to the increase of CVD [11]. The ADT treatment effect may have been weak in our prostate cancer group as the majority of patients had been treated for less than 3 months at the time of the CT scan. 

The positive correlation between abdominal plaque and age or Framingham risk scores is not surprising [5,6,7]. However, our study suggests CECT can also be used to measure abdominal plaque and can even be compared with non-contrast CT (NCT) measurements.

This study has several limitations. The Cases dataset was relatively small; therefore, small differences with the Controls could be missed. However, small differences are not likely to be clinically significant. Our findings warrant confirmation in a larger dataset. As mentioned above, there are other possible confounding factors besides age and gender between the Cases and Controls. Although the age was matched and the Framingham risk scores were not significantly different, known confounding risk factors such as obesity and cholesterol were not separately corrected, and this may have affected the plaque scores. Agatston score measurements can also be influenced by several other factors, including scanner type, tube current, voltage, kernel, and slice thickness [12,13,14,15,16]. In this study, the tube voltages were all 120 kVp, and the slice thickness was identical for all datasets, but other factors were not matched, and these factors may have an effect on the Agatston scores. Although the CT colonography group was used as the non-cancer control group for this study, undiagnosed prostate cancer may be a possibility, as prostate cancer is suggested to be a slowly developing disease with a long preclinical phase [17]. In long-term follow-up, however, only 2 of the control patients were diagnosed with prostate cancer.

## 5. Conclusions

In conclusion, the atherosclerotic plaques of prostate cancer patients in arteries more proximal to the prostate were more correlated with CVD risk factors than with the diagnosis of prostate cancer or prostate cancer-related biomarkers. The presence of abdominal aortic and iliac plaques were highly correlated with each other, suggesting that future studies could avoid the need to measure both arteries. Abdominal arterial plaque scores on CT may serve as indicators for further investigations of CVD when coronary artery calcium scores are unavailable.

## Figures and Tables

**Table 1:** Patient characteristics of the datasets.

	Cases Dataset (*n* = 93)	Controls Dataset (*n* = 186)
Age	63 (IQR, 57–70)	62 (IQR, 58–70)
Gender	Male 100%	Male 100%
Framingham score	11 ± 5.3 (mean ± 2SD)	12 ± 5.2 (mean ± 2SD)
Prior CVD event	7 (7.5%)	21 (11.3%)
PSA	16.02 (IQR, 6.5–61.1)	-
Gleason score	6(3 + 3) 4.3%, 7(3 + 4) 24.7%, 7(4 + 3) 2.2%, 8(3 + 5) 2.2%, 8(4 + 4) 17.2%, 8(unknown individual score) 2.2%, 9(4 + 5) 41.9%, 9(5 + 4) 2.2%, 10(5 + 5) 3.2%	-
Mortality	8.6%	-
Bone metastasis	Present (54.8%), Absent (45.2%)	-
ADT use	Used (54.8%), Not used (45.2%)	-
ADT use term	3 (IQR, 2–5) months	-
Tumor location	PZ (34.4%), TZ (7.5%), PZ + TZ (9.7%), Unknown (49.5%)	-
Prostatectomy	24 (25.8%)	-
Time after prostatectomy	39 months (IQR, 8–88)	-

The “Unknown” categories refer to patients with unavailable pathologic slides or MRI reports. The Cases dataset consists of patients with prostate cancer. The Controls dataset consists of the asymptomatic patients scanned by CT colonography for colorectal cancer screening. Abbreviations: IQR, interquartile range; PSA, prostate-specific antigen at the time of diagnosis; Gleason score, Gleason score from specimen; Mortality, mortality after 23 ± 16 months of follow up; Bone metastasis, bone metastasis after 23 ± 16 months of follow up; ADT use, androgen deprivation therapy use at the time of CT scan; Tumor location, anatomical location of the tumor within the prostate gland; PZ, peripheral zone; TZ, transition zone; Prostatectomy, number of patients who have had prostatectomy at the time of the CT scan; Time after prostatectomy, time between prostatectomy and CT scan.

**Table 2:** Parameters and methods of CT scans.

	Cases Dataset	Controls Dataset
Original reconstruction	1 mm ST (0.8 pitch) Soft kernel (BR40d)	1.25 mm (0.8 pitch) Standard-plus
Resampled for Agatston measurement	3 mm ST	3 mm ST
Tube voltage	120 kVp	120 kVp
Tube current	Automated tube current modulation	30–300 mA
Time delay technique	Fixed time delay (80 s after injection)	-
Scanner type	SIEMENS SOMATOM Force SIEMENS SOMATOM Definition Flash TOSHIBA Aquilion ONE	GE Lightspeed series
Channel	8- to 128-MDCT	16- to 64-MDCT

Abbreviations: ST, section thickness, MDCT, multidetector computed tomography.

**Table 3:** Correlation of abdominal aortic and iliac Agatston scores with CVD risk factors for Cases and Controls.

	Agatston Scores of Cases		Agatston Scores of Controls	
	*p*-Value	Spearman’s Rho	*p*-Value	Spearman’s Rho
Total (AA + CIA + IIA) plaques				
Age	<0.001	0.39	<0.001	0.67
Framingham scores	<0.001	0.41	<0.001	0.49
Abdominal aortic (AA) plaques				
Age	<0.001	0.38	<0.001	0.69
Framingham scores	<0.001	0.39	<0.001	0.51
Common iliac artery (CIA) plaques				
Age	0.001	0.35	<0.001	0.58
Framingham scores	0.002	0.35	<0.001	0.39
Internal iliac artery (IIA) plaques				
Age	0.002	0.34	<0.001	0.58
Framingham scores	<0.001	0.38	<0.001	0.37

**Table 4:** Correlation of abdominal aortic and iliac Agatston scores with CVD risk factors: combined dataset of Cases and Controls.

	Univariate Analysis			Multivariate Analysis		
	β	95% CI for β	*p*-Value	β	95% CI for β	*p*-Value
Total (AA + CIA + IIA) plaques						
Age	0.07	(0.06, 0.09)	<0.001	0.06	(0.04, 0.07)	<0.001
Framingham scores	0.17	(0.12, 0.21)	<0.001	0.08	(0.03, 0.13)	0.002
Prostate cancer	−0.01	(−0.31, 0.29)	0.95	-	-	-
Abdominal aortic (AA) plaques						
Age	0.08	(0.06, 0.09)	<0.001	0.06	(0.04, 0.08)	<0.001
Framingham scores	0.18	(0.13, 0.23)	<0.001	0.09	(0.04, 0.15)	<0.001
Prostate cancer	−0.24	(−0.56, 0.09)	0.16			
Common iliac artery (CIA) plaques						
Age	0.07	(0.06, 0.09)	<0.001	0.06	(0.04, 0.08)	<0.001
Framingham scores	0.15	(0.09, 0.21)	<0.001	0.06	(0, 0.12)	0.057
Prostate cancer	−0.06	(−0.41, 0.28)	0.72			
Internal iliac artery (IIA) plaques						
Age	0.06	(0.05, 0.08)	<0.001	0.05	(0.04, 0.07)	<0.001
Framingham scores	0.13	(0.08, 0.18)	<0.001	0.05	(0, 0.1)	0.064
Prostate cancer	−0.06	(−0.36, 0.24)	0.68			

Correlation of the Agatston scores with age, Framingham risk scores, and prostate cancer diagnosis. The Agatston scores include both the prostate cancer patients and CT colonography outpatient plaques measured in the abdominal aorta, common iliac, and internal iliac arteries. β (unstandardized beta) represents the slope of the line between the Agatston scores and the independent factors. Only factors that had a p-value lower than 0.05 were put into the multivariate analysis. Abbreviations: CI, confidence interval; CVD, cardiovascular disease.

**Table 5:** Association of prostate cancer-related biomarkers and abdominal aortic and iliac Agatston scores in Cases dataset.

	Univariate Analysis				
	β	95% CI for β	*p*-Value	Method	*p*-Value
PSA	−0.00	(−0.00, 0.00)	0.43	Spearman’s correlation	0.73
Gleason score	0.08	(−0.16, 0.33)	0.50	Spearman’s correlation	0.36
Mortality	−0.35	(−1.22, 0.53)	0.43	Mann–Whitney test	0.36
Bone metastasis	0.15	(−0.34, 0.65)	0.55	Mann–Whitney test	0.62
ADT use	−0.00	(−0.50, 0.49)	0.98	Mann–Whitney test	0.98
Tumor location	−0.25	(−0.67, 0.17)	0.24	Kruskal–Wallis test	0.43
Prostatectomy	0.00	(−0.55, 0.57)	0.98	Mann–Whitney test	0.72

The Agatston scores were measured on the abdominal aorta, common iliac, and internal iliac artery plaques. The prostate cancer-related biomarkers include PSA (prostate-specific antigen at the time of diagnosis), Gleason score (Gleason score from specimen), mortality (after 23 ± 16 months of follow up), bone metastasis (after 23 ± 16 months of follow up), ADT use (androgen deprivation therapy use at the time of CT scan), tumor location (the anatomical location of the tumor within the prostate gland), and prostatectomy (patients who have had prostatectomy at the time of the CT scan). β (unstandardized beta) represents the slope of the line between the Agatston scores and the independent factors. Multivariate analysis was not done due to non-significant *p*-values on univariate analysis. Abbreviations: CI, confidence interval.

## Supplementary Materials

The following supporting information can be downloaded at: https://www.mdpi.com/article/10.3390/tomography8020050/s1, Figure S1: Age distribution between prostate cancer patients and the CT colonography outpatients, Figure S2: Residual plot for multivariate analysis in Table 4, Figure S3: Intra-arterial lumen attenuation in portal venous phase CECT of all prostate cancer patients, Figure S4: Flow diagram of calculating conversion factors from multiphase CT scans, Figure S5: Data augmentation of Agatston score measurements using ‘Split islands to segments’ in 3D Slicer, Figure S6: The conversion factor of Agatston scores between non-contrast and portal venous phase CECT in multiphase CT.
