# Supplementary material for: Assessment of Aortoiliac Atherosclerotic Plaque on CT in Prostate Cancer Patients Undergoing Treatment
Source: Tomography. 2022 Mar 1;8(2):607–16. doi: 10.3390/tomography8020050 (PMC8938817; doi:10.3390/tomography8020050)
Supplement: Supplementary file 1 [file tomography-08-00050-s001.zip › Prostate_manuscript_Tomography-Supplementary_final.pdf]

tomography

MDPI

Article

Assessment of Aortoiliac Atherosclerotic Plaque on CT in Prostate Cancer Patients Undergoing Treatment

Supplementary Materials

1. Threshold for measuring plaque in contrast-enhanced CT

To compare plaques in CECT with those on NCT, we needed to first determine a threshold of CT attenuation. To do this, we measured three ROIs (Region of interest) in the intraarterial lumen (ascending aorta, diaphragm level of descending aorta, and aortic bifurcation) in all scans of the Cases dataset. A histogram of the CT attenuations can be found in fig. S3. The mean attenuation was 175±33.2 HU. We chose 250 HU (98-percentile point) as the minimum threshold of plaque since calcified plaques are known to range from 295 HU [1], and also because we expected plaque measurements higher than at least 250 HU would be less contaminated by contrast material in the lumen of PVP and would be easily segmented.

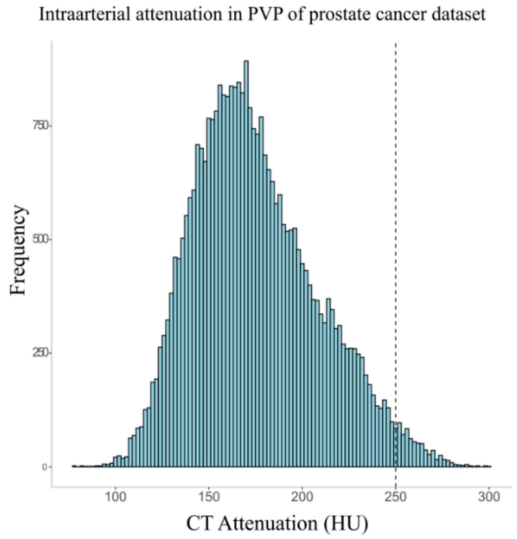

Figure S3. Histogram of intraarterial lumen CT attenuation in the Cases dataset. The CT attenuations ranged from 78 to 300 HU with mean of 175±33.2 HU. The dotted line at 250 HU is at the 98th percentile and served as the threshold for measuring plaques on CECT.

Abbreviations: Hounsfield Unit (HU)

2. Calculating conversion factors

2-1. Introduction

The standard method of measuring an atherosclerotic plaque is the Agatston score [2], which requires NCT. However, an abdominal NCT provides limited medical information for patients and is not scanned routinely to reduce radiation dose [3,4]. Many institutes including our own, opt for a single PVP scan for their routine abdomen-pelvic CT protocol. Thus, plaque measurement on a PVP CECT would be practical. However, Agatston score measurements in CECT require a higher threshold than the conventional 130 HU and are also overestimated because the voxels at the edge of the plaque are averaged with high-attenuation voxels of the lumen, a phenomenon known as partial volume averaging [5]. Coronary artery plaques have been measured on CECT using high thresholds (320HU [6], 600HU [7]), patient-specific thresholds [8-10], and conversion factors [8]. The use of a conversion factor on abdominal plaque measured in CECT has proven to show a high correlation with plaque measured in NCT [11]. Here we use a multiphase CT dataset to calculate the conversion factor of abdominal plaque volume between PVP and NCT. A flow diagram is shown in fig. S4.

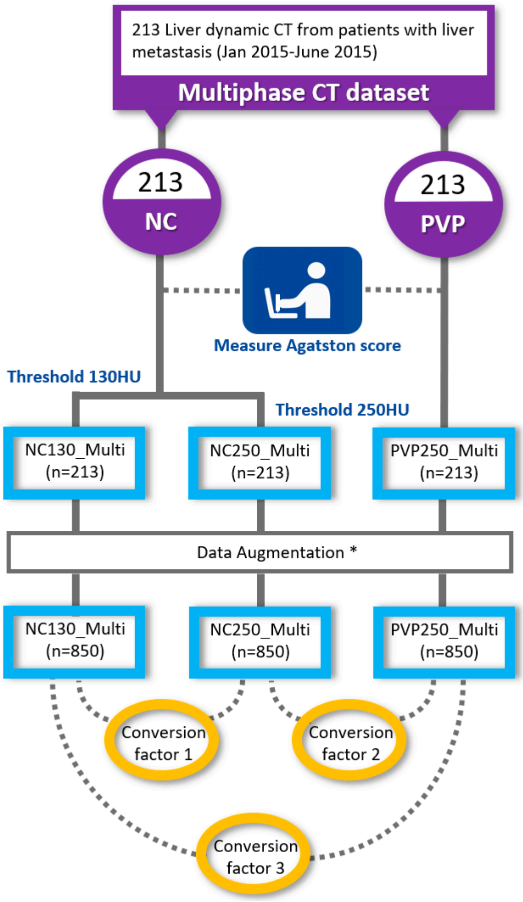

Figure S4. Flow diagram of calculating conversion factors from Multiphase CT scans.

Data Augmentation (*) is described in Figure S5.

Abbreviations: NC, Non-contrast; PVP, Portal venous phase; HU, Hounsfield Unit; NC130_Multi, Agatston scores measured in non-contrast exams of multiphase CT with a threshold of 130 HU; NC250_Multi, Agatston scores measured in non-contrast exams of multiphase CT with a threshold of 250 HU; PVP250_Multi, Agatston scores measured in the portal venous phase of multiphase CT with a threshold of 250 HU; Conversion factor 1, Conversion factor between Agatston scores of 130 HU threshold and 250 HU thresholds (demonstrates the effect of different thresholds on Agatston scores); Conversion factor 2, Conversion factor between Agatston scores of non-contrast exams and portal venous phase exams measured with the same threshold (demonstrates the effect of partial volume averaging due to contrast material); Conversion factor 3, Conversion factor between Agatston scores of non-contrast exams with 130 HU threshold and Agatston scores of portal venous phase exams with 250 HU threshold

2-2. Methods

Dataset

213 multiphase CT scans were collected from a consecutively scanned list of liver multiphase CT scans performed to evaluate liver metastasis at one institution from January to June 2015. The original multiphase CT scans had three phases including non-contrast (NC), arterial phase, and portal venous phase (PVP). NC and PVP were cropped to have the same field of view (FOV) from the diaphragm to the pelvic brim and resampled to 3mm section thickness. The median age was 60 (IQR, 54-66) with males 60.3%. CT parameters are in Table S1.

	**Multiphase CT: Non-contrast**	**Multiphase CT: Portal venous phase**
Original reconstruction	5 mm (0.8 pitch)<br>Soft kernel (BR40d)	2 mm (0.8 pitch)<br>Soft kernel (BR40d)
Resampled for Agatston measurement	3 mm ST	3 mm ST
Tube voltage	120 kVp	120 kVp
Tube current	Automated tube current modulation	Automated tube current modulation
Time delay technique	-	Automatic Bolus Tracking (80-82 seconds after injection)
Scanner type	SIEMENS SOMATOM Force<br>TOSHIBA Aquilion ONE	SIEMENS SOMATOM Force<br>TOSHIBA Aquilion ONE

Table S1. CT parameters of Multiphase CT dataset

Abbreviations: ST, Section thickness

Agatston measurement

A threshold of 130 HU was used to measure the 213 non-contrast exams in the multiphase CT dataset (NC130_Multi). A threshold of 250 HU was used to measure the Agatston scores of 213 PVP CECT in the multiphase CT dataset (PVP250_Multi), and 213 non-contrast exams in the multiphase CT dataset (NC250_Multi). All plaques were measured in the same method by the same tool and person as the prostate cancer dataset and CTC dataset.

Conversion factor calculation

To compensate for the unevenly distributed scores of the multiphase CT measurements, we augmented the 'PVP250_Multi', 'NC250_Multi', and 'NC130_Multi' by splitting the plaques into smaller islands depending on their continuity using the 'Split islands to segments' function in 3D Slicer (version 4.10 RRID: SCR_005619). This process is explained in Fig. S5. The whole plaque (green segmentation in the top row of Fig. S5) is separated into multiple smaller islands (multi-colored segmentations in the bottom row of Fig. S5). Agatston scores of the separated smaller islands are measured and added to the data for data augmentation (multi-colored circles in the bottom row graph of Fig. S5). After the augmentation, a total of 850 sets of measurements were ready to calculate the conversion factors.

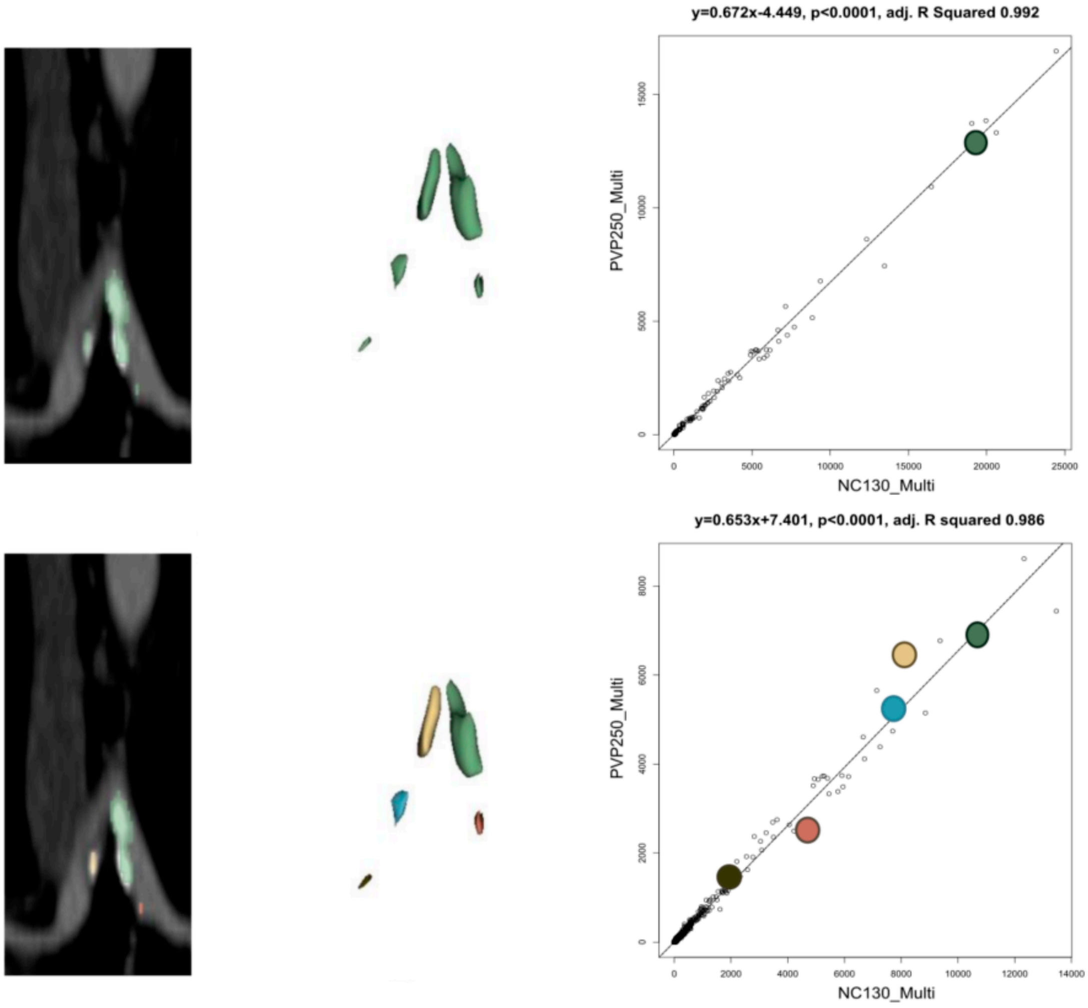

Figure S5. Data augmentation of Agatston score measurements using 'Split islands to segments' in 3D Slicer.
Coronal view images of common iliac artery plaques (first column) and their 3-dimensional reconstructed images (middle column) are shown. Regression lines between the Agatston scores of PVP250_Multi and NC130_Multi are shown in the right column. The colored dot represents the score of the chunk of plaque of the same color from the middle column.

Abbreviations: PVP250_Multi, Agatston scores measured in the portal venous phase of multiphase CT with a threshold of 250 HU; NC130_Multi, Agatston scores measured in non-contrast exams of multiphase CT with a threshold of 130 HU

To calculate the effect of different thresholds on the Agatston scores, we used the 'NC130_Multi' and 'NC250_Multi' scores to draw a linear regression line (conversion factor 1). To calculate the effect of partial volume averaging caused by contrast material in the PVP, we used the 'PVP250_Multi' and 'NC250_Multi' scores (conversion factor 2). To calculate the conversion factor from the traditional Agatston score (measured in NCT with 130 HU) into the Agatston score measured in PVP CECT, we used the 'PVP250_Multi' and 'NC130_Multi' (conversion factor 3). The slopes of the regression lines were used as conversion factors.

Since the conversion factor is derived from the multiphase CT dataset, which is a different dataset from the CTC dataset and the prostate cancer dataset, we decided that it would be reasonable to use this conversion factor only if the intraarterial attenuations of the NC and PVP of the multiphase CT had a mean difference of less than 10 HU with the CTC dataset and prostate cancer dataset, respectively. To confirm this, we randomly sampled 20 scans from each of the prostate cancer datasets, the CTC dataset, the NC, and PVP of the multiphase CT dataset and measured the intraarterial attenuations.

Linear regression was used to calculate the conversion factors between multiphase CT measurements. The slope of the regression line with higher R^2 was used as the conversion factor.

2-3. Result

The conversion factor from 'NC130_Multi' to 'NC250_Multi' was 0.534 (conversion factor 1). The conversion factor from 'PVP250_Multi' to 'NC250_Multi' was 0.808 (conversion factor 2). The conversion factor from 'NC130_Multi' to 'PVP250_Multi' was 0.655 (conversion factor 3). The adjusted R squared were higher than 0.98 in all the regression lines. The regression lines derived from the multiphase CT measurements are shown in Figs. S6c-e.

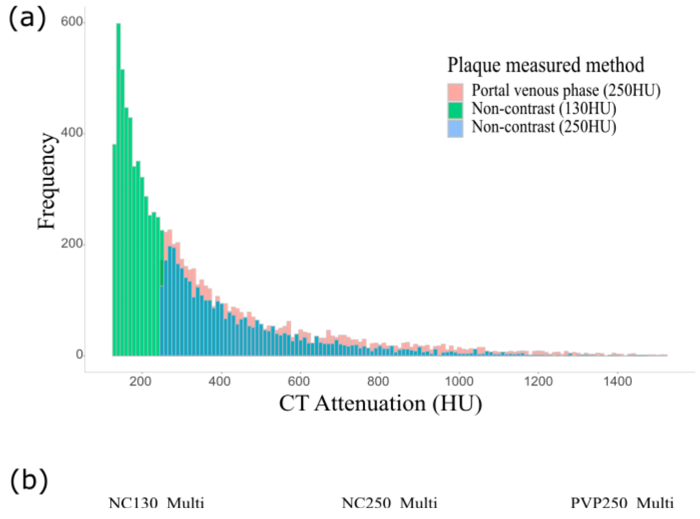

Figure S6. The conversion factor of Agatston scores between non-contrast and portal venous phase CECT in multiphase CT
(a) Histogram of the plaque attenuations measured in multiphase CT. Green bars represent the plaques measured in non-contrast CT with a threshold of 130 HU (NC130_Multi). Blue bars represent the plaques measured in non-contrast CT with a threshold of 250 HU (NC250_Multi). Pink bars represent the plaques measured in portal venous phase CT with a threshold of 250 HU (PVP250_Multi). The median HU values are 236 (IQR, 169-367) for NC130_Multi, 386 (IQR, 302-534) for NC250_Multi, and 403 (IQR, 307-604) for PVP250_Multi.
(b) A 66-year-old man with liver metastasis. Example of plaque segmentation with three different methods. The green area represents NC130_Multi (Plaque area: 33 voxels). The blue area represents NC250_Multi (Plaque area: 23 voxels). The pink area represents PVP250_Multi (Plaque area: 29 voxels).

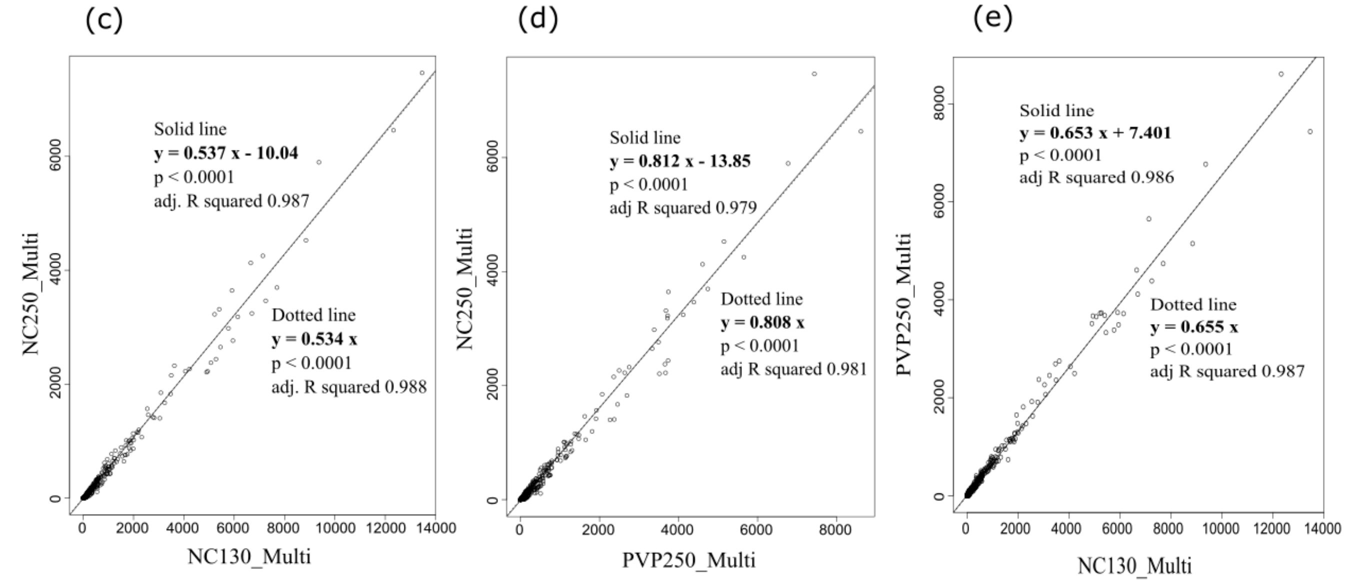

(c)-(e), Linear regression lines between Agatston scores measured in 850 sets of multiphase CT scans. The solid line represents the normal linear regression line with an intercept. The dotted line represents the regression line forced through zero. **(c)** Linear regression line between NC130_Multi scores and NC250_Multi scores. The slope was used for conversion factor 1 (demonstrates the effect of different thresholds on Agatston scores).
(d) Linear regression line between PVP250_Multi scores and NC250_Multi scores. The slope was used for conversion factor 2 (demonstrates the effect of partial volume averaging due to contrast material).
(e) Linear regression line between NC130_Multi scores and PVP250_Multi scores (Conversion factor 3). The slope was used for conversion factor 3 (demonstrates both effects of conversion factors 1 and 2).
Abbreviations: NC130_Multi, Agatston scores measured in non-contrast exams of multiphase CT with a threshold of 130 HU; NC250_Multi, Agatston scores measured in non-contrast exams of multiphase CT with a threshold of 250 HU; PVP250_Multi, Agatston scores measured in the portal venous phase of multiphase CT with a threshold of 250 HU; IQR, interquartile range

The median intraarterial attenuations of the NC multiphase CT and CTC dataset were 40 (IQR, 38-43) and 45 (IQR, 42-49). The median intraarterial attenuations of the PVP multiphase CT and prostate cancer CT were 174 (IQR, 165-185) and 174 (IQR, 158-198). Therefore, we determined the conversion factors were appropriate to use on the Case and Controls datasets.

2-4. Discussion

The conversion factor between NCT and CECT in our study was 0.655 (conversion factor 3) which was comparable to the conversion factor between NCT and CECT of a renal donor dataset

measured with a deep learning-based fully automated Agatston scoring model (0.6866) [11]. There have been other studies reporting conversion factors of 0.46 (1/2.19) [12] and 0.19 (1/5.016) [10] between NCT and CECT plaque scores of CT coronary angiography. The scores in these studies are measured with a patient-specific threshold due to the extremely high intraluminal contrast media in angiography, and thus cannot be directly compared to our study.

Each conversion factor in this study has a meaning and here we attempt to explain them in more detail. With conversion factor 1, we have demonstrated that increasing the threshold from 130 HU to 250 HU led to a decrease in plaque area (green and blue segmentation in Fig. S6b) causing a decrease in the Agatston score by approximately 53.4% (slope of the dotted line in Fig. S6c). However, even when the same threshold of 250 HU is used on both NCT and CECT, there was still a difference in the segmentation (blue and pink segmentation in Fig. S6b) and attenuation histogram (blue and pink bars in Fig. S6a). This is largely due to partial volume averaging [13] at the margin of the plaque that is adjacent to the high attenuation contrast media. With conversion factor 2, we have demonstrated that partial volume averaging in the PVP caused an overestimation of the Agatston score by approximately 24% (PVP250_Multi is 1/0.808 % of NV250_Multi, 0.808 is the slope of the dotted line in Fig. S6e). Conversion factor 3 would correct both the effect of threshold and partial volume averaging, and may be used to directly convert plaque scores measured in PVP CT scans with 250 HU into scores measured in NCT with a traditional method of 130 HU.

In the main manuscript, 0.808 (conversion factor 2) was used to calibrate the Cases group Agatston scores and compare them with the Controls group scores. We measured the Agatston score of CTC with a threshold of 250 HU and used conversion factor 2 (instead of measuring with 130 HU and using conversion factor 3) in order to eliminate any possibility of noise artifact in the low dose CTC.

Reference

1. Sun, J.; Zhang, Z.; Lu, B.; Yu, W.; Yang, Y.; Zhou, Y.; Wang, Y.; Fan, Z. Identification and quantification of coronary atherosclerotic plaques: a comparison of 64-MDCT and intravascular ultrasound. *AJR Am J Roentgenol* **2008**, *190*, 748-754, doi:10.2214/ajr.07.2763.
2. Agatston, A.S.; Janowitz, W.R.; Hildner, F.J.; Zusmer, N.R.; Viamonte, M., Jr.; Detrano, R. Quantification of coronary artery calcium using ultrafast computed tomography. *J Am Coll Cardiol* **1990**, *15*, 827-832, doi:10.1016/0735-1097(90)90282-t.
3. Goo, H.W. CT radiation dose optimization and estimation: an update for radiologists. *Korean J Radiol* **2012**, *13*, 1-11, doi:10.3348/kjr.2012.13.1.1.
4. Rastogi, S.; Singh, R.; Borse, R.; Zujic, P.V.; Segota, D.; Diklic, A.; Jurkovic, S.; Ali, A.; Kharita, H.M.; Al-Naemi, H.M.; et al. Use of Multiphase CT Protocols in 18 Countries: Appropriateness and Radiation Doses. *Can Assoc Radiol J* **2020**, 846537119888390, doi:10.1177/0846537119888390.
5. Buijs, R.V.C.; Leemans, E.L.; Greuter, M.; Tielliu, I.F.J.; Zeebregts, C.J.; Willems, T.P. Quantification of abdominal aortic calcification: Inherent measurement errors in current computed tomography imaging. *PLoS One* **2018**, *13*, e0193419, doi:10.1371/journal.pone.0193419.
6. Otton, J.M.; Lønborg, J.T.; Boshell, D.; Feneley, M.; Hayen, A.; Sammel, N.; Sesel, K.; Bester, L.; McCrohon, J. A method for coronary artery calcium scoring using contrast-enhanced computed tomography. *J Cardiovasc Comput Tomogr* **2012**, *6*, 37-44, doi:10.1016/j.jcct.2011.11.004.
7. Glodny, B.; Helmel, B.; Trieb, T.; Schenk, C.; Taferner, B.; Unterholzner, V.; Strasak, A.; Petersen, J. A method for calcium quantification by means of CT coronary angiography using 64-multidetector CT: very high correlation with Agatston and volume scores. *Eur Radiol* **2009**, *19*, 1661-1668, doi:10.1007/s00330-009-1345-2.
8. Bischoff, B.; Kantert, C.; Meyer, T.; Hadamitzky, M.; Martinoff, S.; Schömig, A.; Hausleiter, J. Cardiovascular risk assessment based on the quantification of coronary calcium in contrast-enhanced coronary computed tomography angiography. *Eur Heart J Cardiovasc Imaging* **2012**, *13*, 468-475, doi:10.1093/ejechocard/jer261.
9. Mylonas, I.; Alam, M.; Amily, N.; Small, G.; Chen, L.; Yam, Y.; Hibbert, B.; Chow, B.J. Quantifying coronary artery calcification from a contrast-enhanced cardiac computed tomography angiography study. *Eur Heart J Cardiovasc Imaging* **2014**, *15*, 210-215, doi:10.1093/ehjci/jet144.
10. Schuhbaeck, A.; Otaki, Y.; Achenbach, S.; Schneider, C.; Slomka, P.; Berman, D.S.; Dey, D. Coronary calcium scoring from contrast coronary CT angiography using a semiautomated standardized method. *J Cardiovasc Comput Tomogr* **2015**, *9*, 446-453, doi:10.1016/j.jcct.2015.06.001.
11. Summers, R.M.; Elton, D.C.; Lee, S.; Zhu, Y.; Liu, J.; Bagheri, M.; Sandfort, V.; Grayson, P.C.; Mehta, N.N.; Pinto, P.A.; et al. Atherosclerotic Plaque Burden on Abdominal CT: Automated Assessment With Deep Learning on Noncontrast and Contrast-enhanced Scans. *Acad Radiol* **2020**, doi:10.1016/j.acra.2020.08.022.
12. Pavitt, C.W.; Harron, K.; Lindsay, A.C.; Zielke, S.; Ray, R.; Gordon, D.; Rubens, M.B.; Padley, S.P.; Nicol, E.D. Technical feasibility and validation of a coronary artery calcium scoring system using CT coronary angiography images. *Eur Radiol* **2016**, *26*, 1493-1502, doi:10.1007/s00330-015-3940-8.
13. van der Bijl, N.; Joemai, R.M.; Geleijns, J.; Bax, J.J.; Schuijf, J.D.; de Roos, A.; Kroft, L.J. Assessment of Agatston coronary artery calcium score using contrast-enhanced CT coronary angiography. *AJR Am J Roentgenol* **2010**, *195*, 1299-1305, doi:10.2214/ajr.09.3734.
