# Supplementary figures and images for: Assessment of Aortoiliac Atherosclerotic Plaque on CT in Prostate Cancer Patients Undergoing Treatment
Source: Tomography. 2022 Mar 1;8(2):607–16. doi: 10.3390/tomography8020050 (PMC8938817; doi:10.3390/tomography8020050)

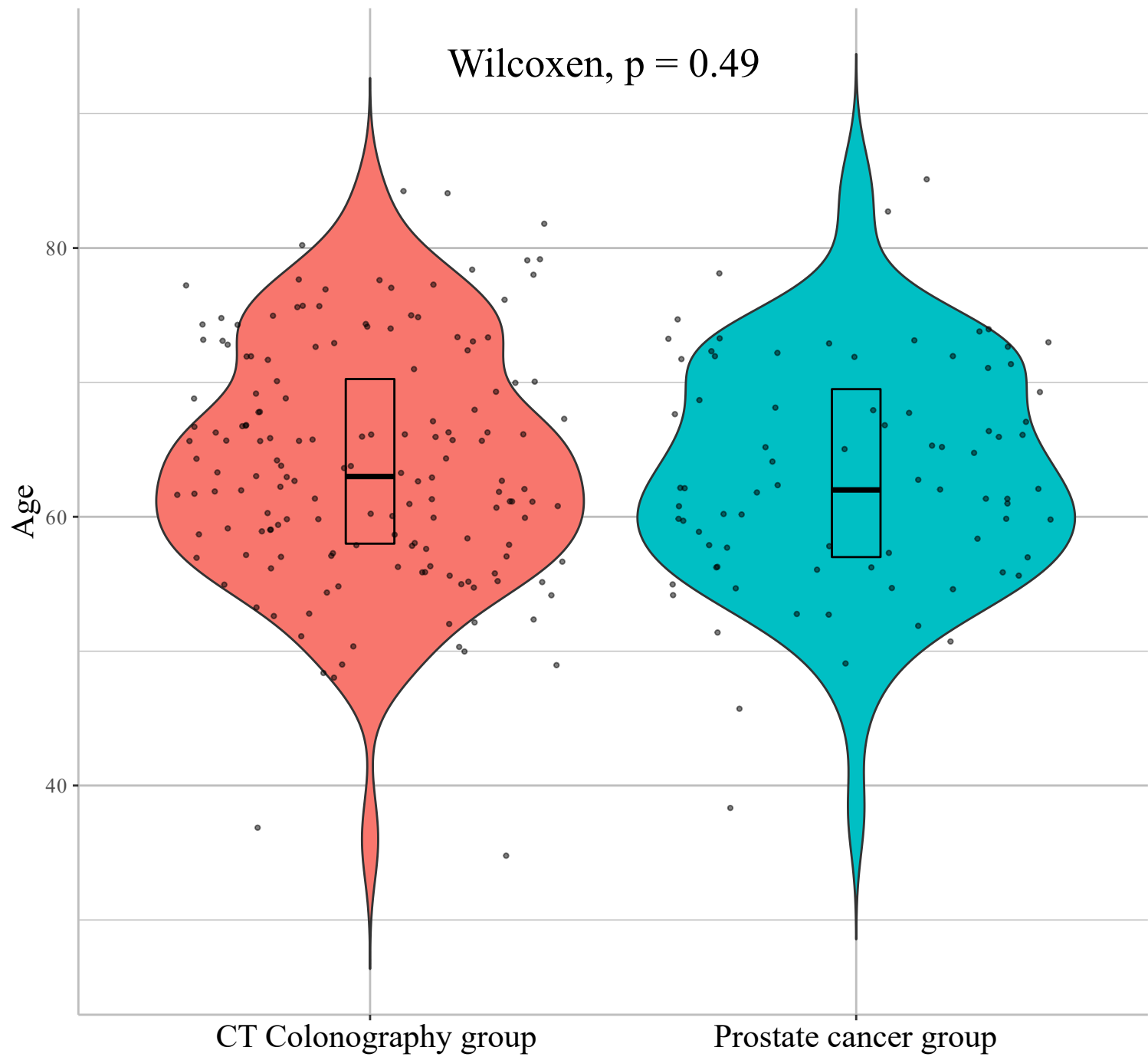

Supplement: Supplementary file 1 [file tomography-08-00050-s001.zip › SFig1_Age_CTCvsPcan_violinplot.pdf]

**Residual plot**

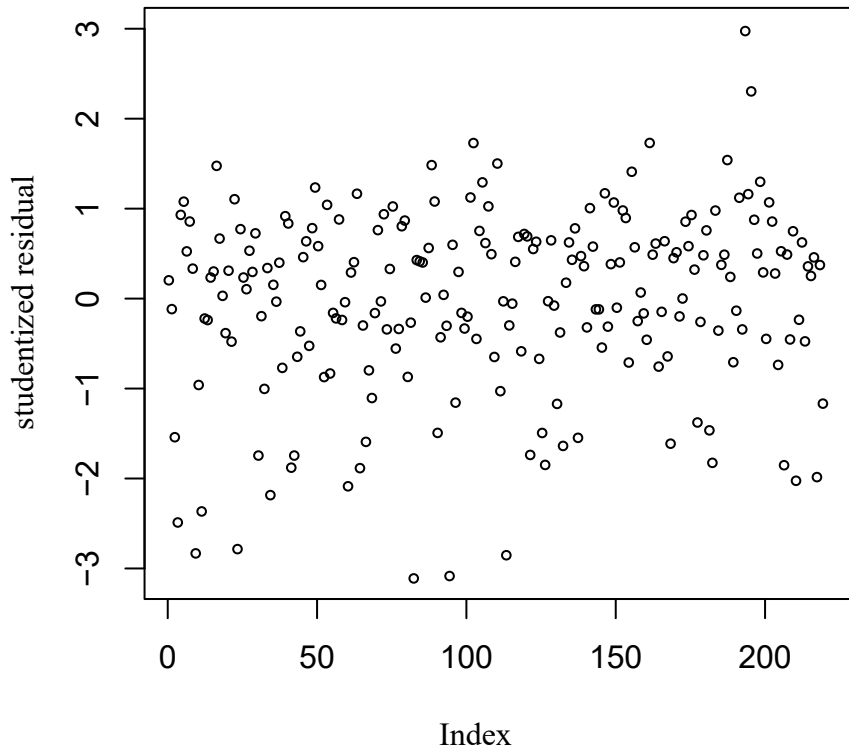

Supplement: Supplementary file 1 [file tomography-08-00050-s001.zip › sFig2_residuals.pdf]

# Intraarterial attenuation in PVP of prostate cancer dataset

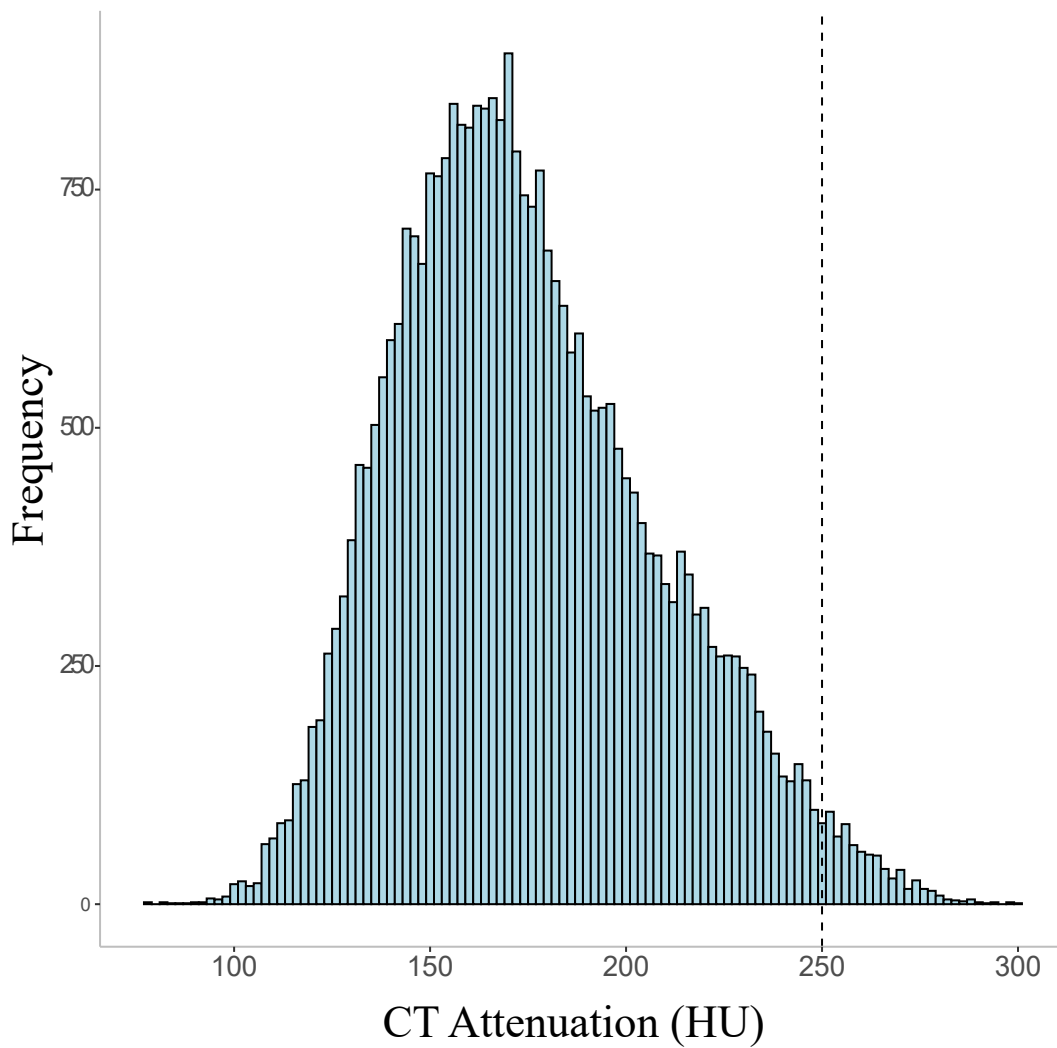

Supplement: Supplementary file 1 [file tomography-08-00050-s001.zip › SFig3_Allvessel_hist.pdf]

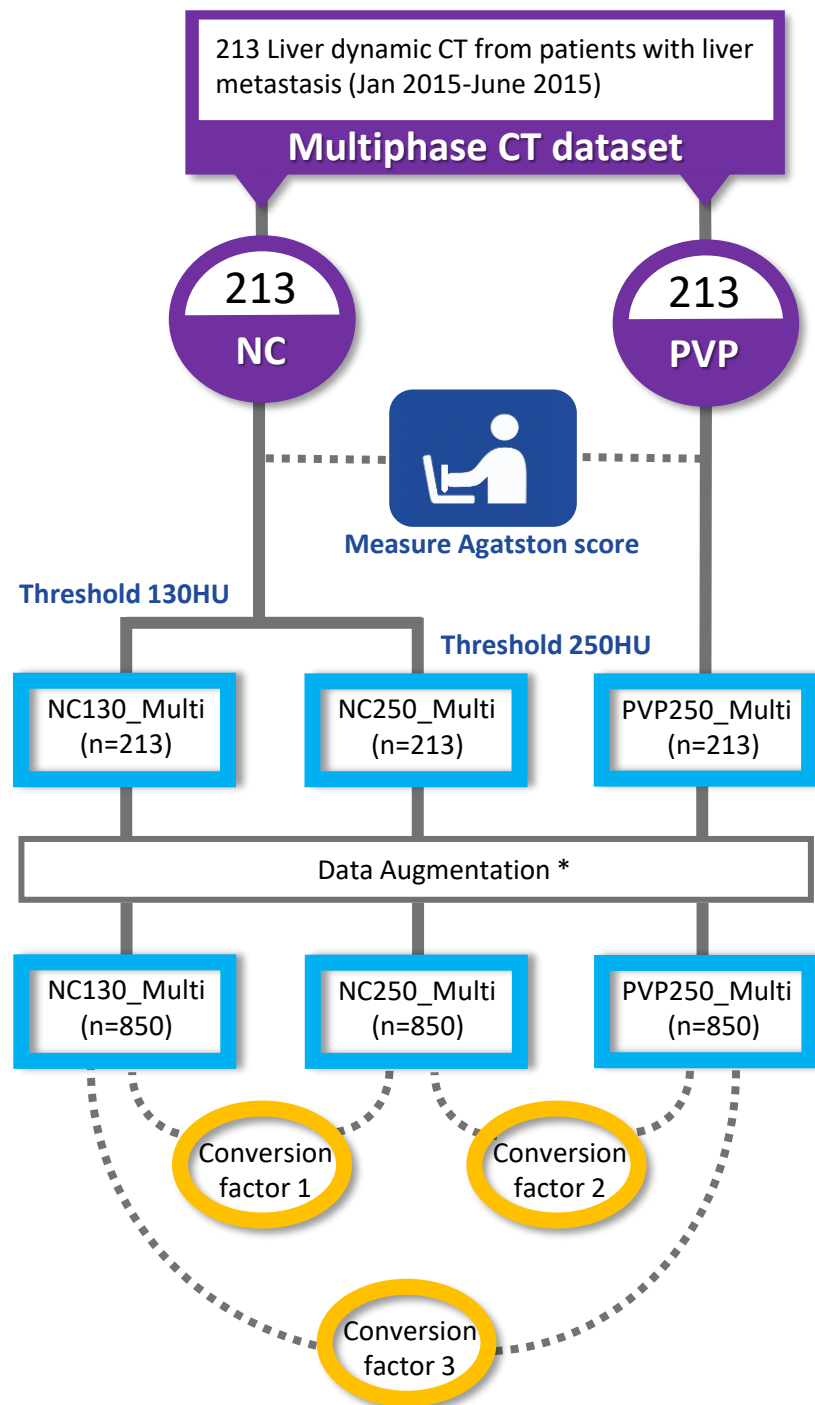

Supplement: Supplementary file 1 [file tomography-08-00050-s001.zip › SFig4_STARD_conversionfactor.pdf]

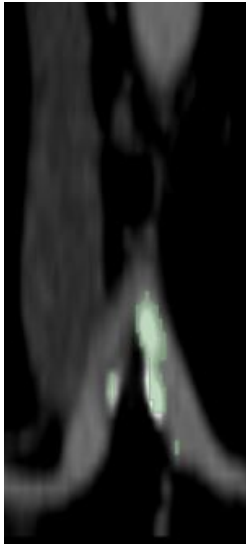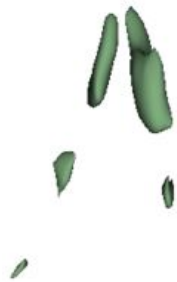

$y=0.672x-4.449$ ,  $p<0.0001$ , adj. R Squared 0.992

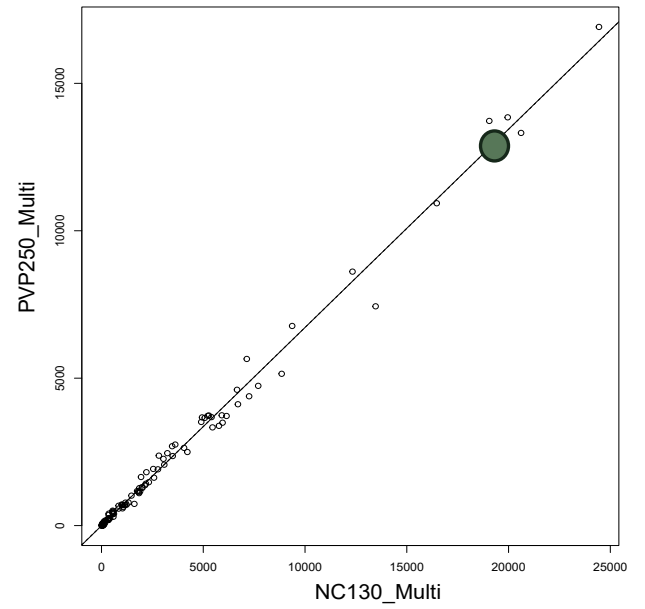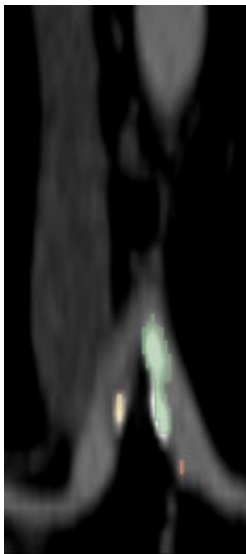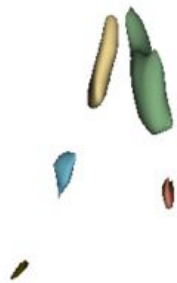

$y=0.653x+7.401$ ,  $p<0.0001$ , adj. R squared 0.986

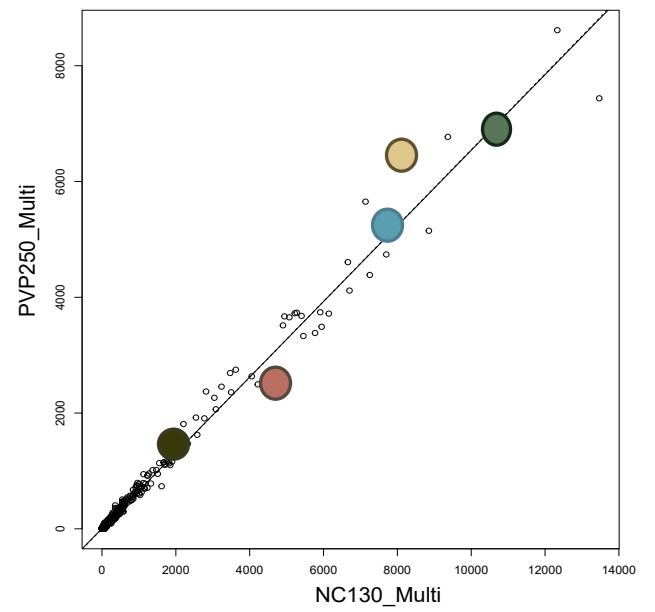

Supplement: Supplementary file 1 [file tomography-08-00050-s001.zip › sFig5_Augmentation.pdf]

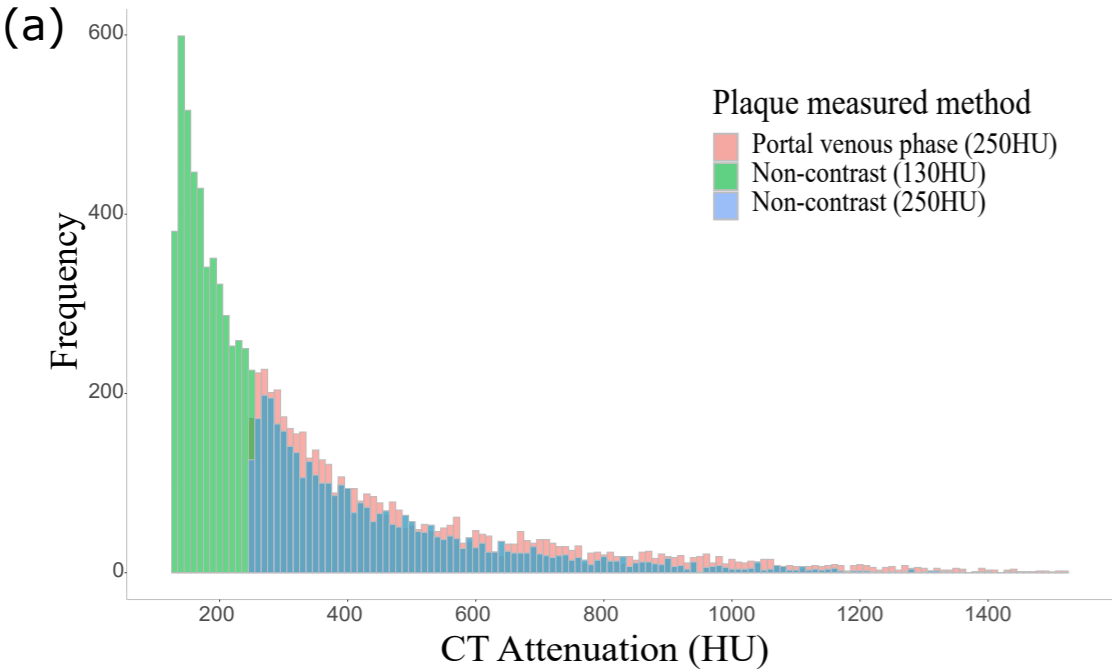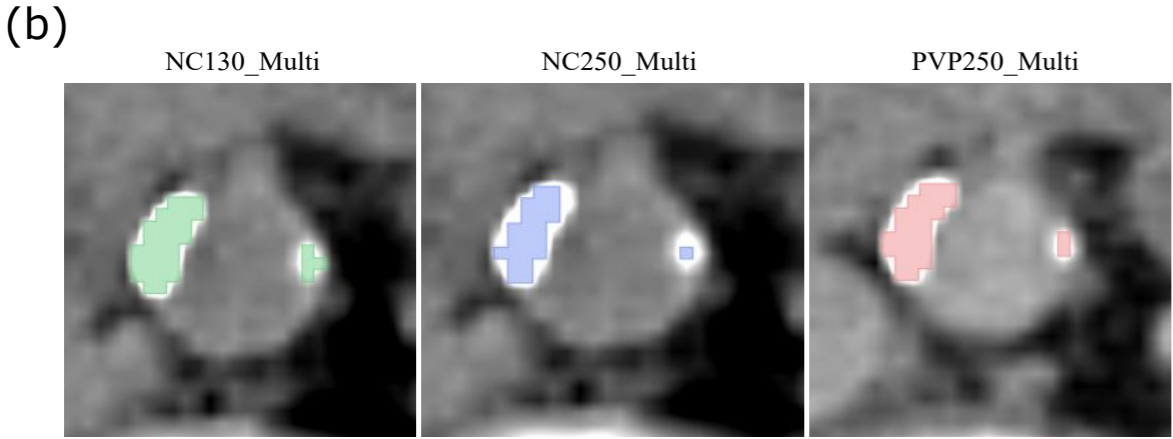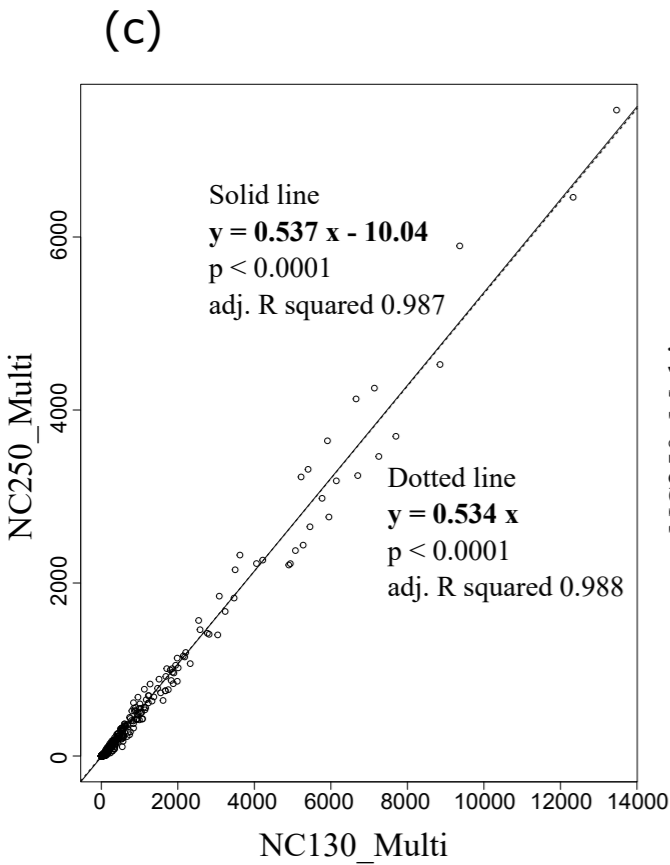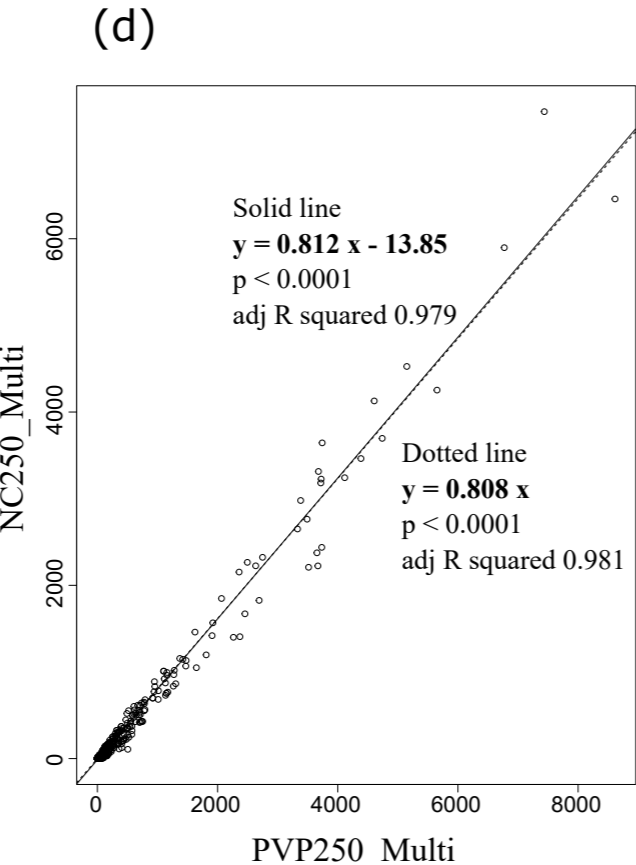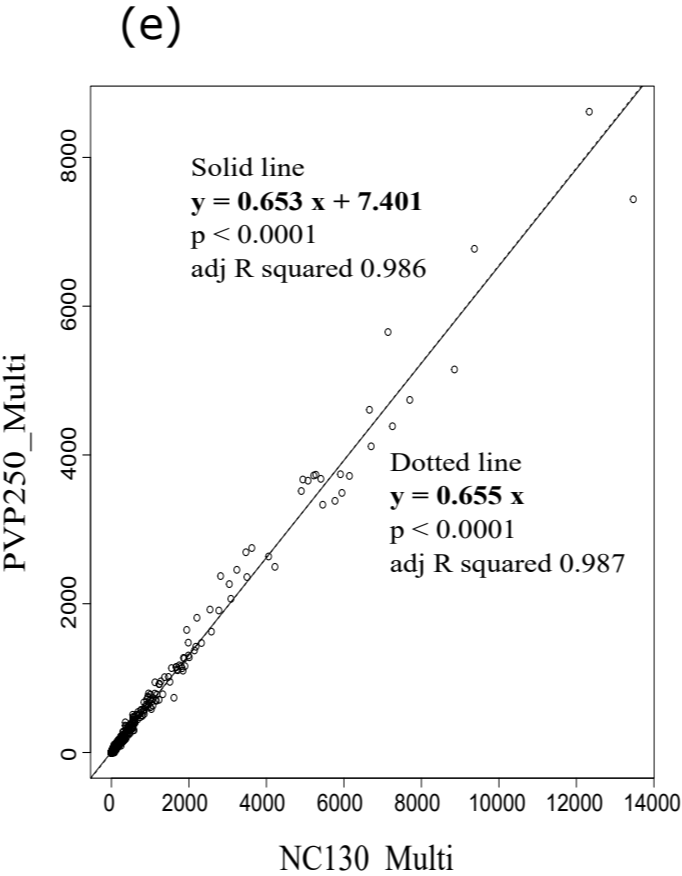

Supplement: Supplementary file 1 [file tomography-08-00050-s001.zip › sFig6_Conversion factors.pdf]
